# Effect of different dietary inclusion levels of whole plant green tomato (*Physalis philadelphica*) silage on nutrient intake and digestibility, and *in vitro* rumen fermentation kinetics in sheep

**DOI:** 10.3389/fvets.2022.980619

**Published:** 2022-10-14

**Authors:** Lizbeth E. Robles-Jimenez, Alondra C. Narváez-López, Alfonso J. Chay-Canul, Aurora Sainz-Ramirez, Octavio A. Castelan-Ortega, Naifeng Zhang, Manuel Gonzalez-Ronquillo, Einar Vargas-Bello-Pérez

**Affiliations:** ^1^Departmento de Producción Animal, Facultad de Medicina Veterinaria y Zootecnia, Universidad Autónoma del Estado de México, Toluca, Mexico; ^2^División Académica de Ciencias Agropecuarias, Universidad Juárez Autónoma de Tabasco, Villahermosa, Mexico; ^3^Institute of Feed Research of Chinese Academy of Agricultural Sciences, Key Laboratory of Feed Biotechnology of the Ministry of Agriculture and Rural Affairs, Beijing, China; ^4^Department of Animal Sciences, School of Agriculture, Policy and Development, University of Reading, Reading, United Kingdom

**Keywords:** green tomatoes, corn silage, sheep, fermentation kinetics, crop residues

## Abstract

Mexico has many agricultural by-products that can be used for animal feed, and green tomatoes are produced throughout the country and can be an alternative to overcome the high prices of cereal-based feeds. This study determined *in vitro* fermentation kinetics, production performance, nutrient intake, digestibility, and nitrogen balance from sheep supplemented with whole plant green tomato (GT) on corn silage (CS) based diets. For 21 days, eighteen Suffolk lambs (38 ± 4 kg of live weight) were grouped into three dietary GT inclusion levels to replace CS: a control diet based on 100% CS (GT0, 570 g /kg dry matter, DM), while 100 g/kg DM (GT100) and 200 g/kg DM (GT200) of GT were included as a replacement for CS. A completely randomized design was used to measure *in vitro* gas production, *in vitro* rumen fermentation, chemical composition, and *in vivo* parameters. *In vitro* gas production, “A” (ml/g DM), fermentation rates “B,” (h^−1^), and “C” (h^−½^), were lower for GT200, while DM disappearance (mg/100mg) was lower for GT100 compared with GT0. Compared to GT0, GT100 and GT200 did not affect (*P* > 0.05) DM and organic matter (OM) intake (g/kg^LW0.75^). Ether extract intake was higher for GT0 and GT100 (*P* < 0.001) compared to GT200. Neutral detergent fiber (NDF) intake was higher (P < 0.05) for GT200 compared with GT0. Intake of lignin was higher (*P* < 0.001) for GT200 than that of GT0 and GT100. Digestibility coefficients for DM, OM, NDF, and Acid detergent fiber (ADF) were lower (*P* < 0.05) in GT100 than in the rest of the treatments. Nitrogen intake and N excreted in feces and urine were lower (*P* < 0.001) for GT0. N balance was negative for all treatments, being higher for GT200 (*P* < 0.05). Overall, the addition of GT at 100 or 200 g/kg DM in sheep diets negatively affects nutrient digestibility and N balance, so their dietary inclusion is not recommended.

## Introduction

Mexico is an important producer and exporter of vegetables worldwide and the main vegetables produced are green (*Physalis philadelphica*) and red (*Solanum lycopersicum*) tomatoes. Mexico has many agricultural by-products that can be used for animal feeding, and this is an alternative to overcome the high prices of cereal-based feeds (1). Red tomato by-products such as hay or silage have been used in sheep (1, 2) diets. Forages with high water content can be cut and dried in the field until they reach 35% dry matter (DM) and then ensiled to conserve their nutritional content (3). Green tomato is harvested in almost all-Mexican territory, producing around 7,712 thousand tons per year (4). The importance of green tomatoes lies in their culinary use, and their antioxidant and vitamin contents (5), and when tomatoes are harvested, the plant residues are left on the field and used as organic matter for soil improvement. These by-products could be used as ruminant feed, either as a grazing material or cut fresh (6). To date, there are no reports on the use of green tomato silage for ruminant feed. The hypothesis of this study was that green tomato silage can alternate with corn silage in lamb diets, thus two experiments (*in vitro* and *in vivo*) were conducted to determine the effect of different inclusion levels of whole plant green tomatoes on *in vitro* gas production and rumen fermentation kinetics, nutrient intake, digestibility, and nitrogen balance in sheep.

## Materials and methods

### Green tomatoes and corn crops

Forage samples of whole plant green tomatoes (*Physalis philadelphica*) with fruits were collected at 2,840 m above sea level, between the coordinates 19° 04' north latitude and 99°32' west longitude. The climate is humid temperate, Cb (w2) (w) (i') (g), with summer rains and little thermal oscillation (7). Green tomatoes (GT) seeds were planted in May 2019. A total area of 100 m^2^ was previously fertilized with sheep manure. For the study, the vegetative stage of the plant was left to mature until fruits were harvested at 90 days after cropping. Then, GT whole plant was chopped in a hammer mill (size 5 cm Ø) and left to sun dry for 4 days. In another crop, 100 m^2^ of whole corn plants were harvested when the plant was in a milky stage, and they were chopped using a hammer mill (5 cm Ø) for subsequent corn silage (CS) production. Then, silages from green tomatoes and corn, were placed in layers, compacted, sealed, and ensiled in six hard plastic containers (975 mm in height, 594 mm Ø, and 208 liters of capacity) per treatment (GT Whole Plant or CS Whole Plant), together with the addition of 0.001% fresh Pulque as an inoculant to accelerate the silage fermentation process (8).

### *In vitro* gas production

A buffer solution was prepared according to Menke and Steingass (9), where 200 mg DM of each diet mixture was incubated in glass syringes of 100 mL. Ruminal fluid (300 mL per animal) was obtained from three fistulated male Suffolk sheep ([62 ± 3 kg Live weight (LW) (average ± SD)]), previously fed with the control diet. Ruminal fluid was extracted and filtered through a triple layer of gauze and homogenized with CO_2_ for 5 min. Glass syringes were filled under anaerobic conditions with 200 mL of the previous mixture (100 mL of rumen inoculum and 900 mL of an incubation solution). In 1 L, this solution consisted of 238 mL/L of buffer solution [14 g NaHCO_3_ and 1.5 g (NH4)HCO_3_ per L], 238 mL/L of a macro mineral solution (5.7 g Na_2_HPO_4_, 6.2 g KH_2_PO_4_ and 0.6 g MgSO_4_.7H_2_O per L), 474 mL/L of distilled water, 0.1 mL/L of micro minerals (13.2 g CuCl_2_.2H_2_O, 10.0 g MnCl_2_4H_2_O, 1.0 g CoCl_2_.6H_2_O, 8.0 g FeCl_2_.6H_2_O and made up to 100 mL with H_2_O) and 50 mL/L of a reduction solution (47.5 mL distilled water, 2 mL of 1N NaOH and 313 mg HCl-cysteine), and resazurin (phenoxazine dye). Two additional syringes without substrate were also prepared as blanks to account for the presence of other soluble extracts on overall gas production and to correct readings for substrate, including syringes from the self-fermentation of rumen inoculum. Glass syringes per triplicate per treatment, were filled with the incubation solution under a CO_2_ stream, and incubated for 96 h in a water bath at 39°C. The gas volume was recorded at 3, 6, 9, 12, 24, 36, 48, 60, 72, 84, and 96 h of incubation in three series of incubation.

### Animals and diets

All experimental procedures were approved by the Animal Experimental Guidelines of the Universidad Autonoma del Estado de México (project code UAEMex 4974/2020). Eighteen male Suffolk lambs [38 ± 4 kg LW (average ± SD)], were arranged in a completely randomized design for a 21-day period that consisted of 14 d for diet adaptation and 7 d for sample collection.

The control diet was formulated according to the nutritional requirements for growing sheep (10) receiving 105 g crude protein (CP)/d and 10.5 MJ metabolizable energy/kg DM. Three levels of GT inclusion were used to replace CS: a control diet based on 100% CS (GT0), 100 g/kg DM (GT100), and 200 g/kg DM (GT200) of GT were included as replacements for CS. Chemical composition of feedstuffs and treatments is shown in Tables 1, 2 respectively. Before feeding, individual live weights were measured at the beginning and end of the experimental period. Animals were housed in individual metabolic cages (1.20 × 0.80 m), fed individually twice a day (0800 and 1,500 h), with free access to water. Feed intake and refusals were measured daily but only data from the last 7 days were accounted for the statistical analysis. During the last 7 days of the period, samples of feces and urine were collected daily and 10% of the total samples for feces and urine were frozen at −20°C for further analysis.

**Table 1:** Chemical composition (g/kg DM) of individual feedstuffs.

**Parameter**	**Green tomato silage**	**Corn silage**	**Alfalfa hay**	**Sorghum grain**	**Soya bean meal**	**Canola meal**	**Wheat bran**
Dry matter^1^	319 ± 16	256 ± 13	900 ± 45	900 ± 43	880 ± 42	890 ± 44	900 ± 42
Organic matter	804 ± 40	911 ± 45	910 ± 43	980 ± 49	940 ± 47	900 ± 27	930 ± 37.2
Crude protein	101 ± 4	73 ± 3	140 ± 7	80 ± 3	430 ± 17	320 ± 13	150 ± 7
Ether extract	28 ± 2	32 ± 2	60 ± 3	30 ± 1	23 ± 1	32 ± 1	46 ± 2
Neutral detergent fiber	562 ± 22	496 ± 25	540 ± 27	150 ± 6	150 ± 5	330 ± 13	480 ± 24
Acid detergent fiber	252 ± 10	229 ± 9	390 ± 16	50 ± 2	100 ± 4	190 ± 7	130 ± 5
Acid detergent lignin	77 ± 4	57 ± 2	70 ± 3	30 ± 1	40 ± 2	50 ± 2	60 ± 3
Metabolizable energy^2^	9.4	10.0	8.4	12.6	12.6	10.9	10.5
pH	4.2 ± 0.12	4.1 ± 0.16					
Ammonia N (mg/dl)	16.2 ± 0.6	7.61 ± 0.4					
**Volatile fatty acids (mol/100 mol)**							
Acetic acid	70.3 ± 3	75.7 ± 3					
Propionic acid	17.5 ± 3	12.0 ± 3					
Butyric Acid	12.2 ± 0.6	11.9 ± 0.5					

1 DM, dry matter expressed as fresh matter (g/kg);

2 Expressed as MJ/kg DM.

**Table 2:** Feedstuff inclusion (g/kg DM) and chemical composition (g/kg DM) of dietary treatments.

**Ingredients g/kg**	**GT0^2^**	**GT100**	**GT200**
Corn silage	570	470	370
Green tomato silage	0	100	200
Alfalfa hay	87	87	87
Sorghum grain	235	235	235
Soyabean meal	43	43	43
Canola meal	27	27	27
Wheat bran	22	22	22
Minerals and vitamins^1^	16	16	16
Total	1000	1000	1000
**Chemical composition**			
Dry matter	534	540	546
Organic matter	913	902	891
Crude protein	103	105	108
Ether extract	32	33	32
Neutral detergent fiber	391	397	404
Acid detergent fiber	188	190	193
Lignin	50	52	54
Metabolizable energy, MJ/kg DM	10.5	10.4	10.3

1 Mineral and vitamin supplement, in 1.0 kg DM it contains the following: 25 mg of antioxidant, 4.5 g of calcium carbonate, 6 g of salt, 30 g of ionophore, 50 g of zinc oxide, 6 g of sodium bicarbonate, 6 g of copper sulfate, 20 g of ferrous sulfate, 125 g of sodium sulfate, 18000 IU of vitamin E, 3 000 000 UI of vitamin A, 3 750 000 IU of vitamin D, 140 g of potassium chloride, 0.500 g of E.D.D. I ethylene-dynamine, 0.090 g of cobalt carbonate, 500 mg of magnesium oxide, 36 g of manganese oxide, and 0.090 g of selenium.

2 GT, green tomato silage.

### Chemical analyses

All chemical analyses were performed in triplicate. Feed orts, fecal samples, individual feedstuffs, and diets were dried in a forced air oven (60°C, 48 h), then ground in a mill (Willey, 2 mm Ø Arthur H. Thomas Philadelphia, PA) to determine organic matter (OM; 942.05) (9). Total nitrogen (N; 954.01) was determined by the Kjeldahl method (9) using a conversion factor of 6.25 for crude protein (CP) determination. Neutral detergent fiber (NDF), acid detergent fiber (ADF) and lignin were determined according to Van Soest (11) with the addition of sodium sulfite and alpha-amylase using an ANKOM fiber system. All feedstuffs were analyzed for ether extract (920.39) (12). Composite samples of tomato (*n* = 6) and corn (*n* = 6) silages were analyzed for ammonia and volatile fatty acids (VFA) by gas chromatography according to Moon et al. (13). Corn and green tomato silages pH were determined in triplicate. Feces and urine samples were used for nitrogen (N; 991.20) determination (12) to assess nitrogen excretion. Nutrient digestibility coefficients were determined as: digestibility (g/kg) = (nutrient intake – nutrient excreted) / (nutrient intake) × 1,000 (14).

### Calculations

The accumulated gas volume of each sample was determined using the model proposed by France et al. (15).


(1) $$Y=A\;[1-exp\;(-B(t-T)-C(\sqrt{t}-\frac{A}{T}))]$$

Where: “*Y*” is the cumulative gas production (mL) “*t*” is the incubation time (*h*), A is the asymptote curve (total gas produced, mL), B (*h*^−1^), and C (*h*^−½^) are the gas production constants, T is the time of delay (h) that colonize the microorganisms to begin the fermentation.

After *in vitro* incubation periods, samples were filtered and dried (48 h, 60°C) to determine dry matter disappearance (DMd96h), and gas yield production at 24 h (GY24). The volume of gas (mL gas/g DM) produced after 24 h of incubation was calculated by dividing the amount of DMd96h (g):


(2) $$\begin{array}{l} {Gas\;production\;\left(GY24\right)=mLgas\;24/gDMd96h}_{\;} \end{array}$$

The gas production (GP) at 96 h was correlated with DM disappearance to produce relative gas yield (RGY; mL gas/g DMd96h) (16).

Intakes (kg/day and g/kg LW^0.75^) were estimated during the feeding trial.

### Statistical analysis

Each *in vitro* experiment was completed in 4 days, using three replicates per treatment per incubation run (nine replicates per treatment). The analytical replicates were averaged before statistical analysis, so the statistical number of treatment replicates (*n* = 3) are the true replicates (three incubation runs). The experimental unit was therefore the mean of the three replicates obtained per incubation run forms a statistical replicate. A completely randomized design was used to determine *in vitro* gas production, and *in vitro* microbial fermentation (17). Orthogonal contrast was used to test the linear and quadratic responses of each *in vitro* dependent variable at increasing levels (G0, G100, G200 g/kg DM) of green tomato silage.

A completely randomized design was used to determine the chemical composition and *in vivo* parameters (17) as follows.


(3) $$\begin{array}{l} {Y_{ij}=\mu \;+\;{Tx}_{i}\;+\;\varepsilon_{ij}}_{\;} \end{array}$$

Where *Y*_ij_ = is each observation of treatments it; μ is the general mean; *T*x_i_ (*n* = 3) is the treatment effect; and ε_ij_ is the experimental error.

Data from the *in vivo* experiment (*n* = 18) were analyzed using a one-way ANOVA (18). Where treatment (G0, G100, G200 g/kg DM) was the main effect. Digestibility and N balance data also were analyzed using a one-way ANOVA. The Tukey test was used when significant differences were observed between treatments *P* < 0.05. Orthogonal contrast was used to test the linear and quadratic responses to determine differences among treatments.

## Results

### *In vitro* fermentation kinetics

*In vitro* fermentation parameters ate shown in Table 3. Gas production “A” (mL/g DM), fermentation rates “B” (h^−1^), and “C” (h^−½^), were lower for GT200. DMd96h was lower (*P* < 0.05) for GT100. Gas production at 24 h and RGY were higher (*P* < 0.05) for GT0 than the rest of the treatments.

**Table 3:** *In vitro* gas production from different inclusion levels of whole green tomato silage (GT) as an alternative to corn silage.

	**Treatment**			**SEM**	***P*** **-value**		
	**GT0**	**GT100**	**GT200**		**Treatment**	**Lineal**	**Quadratic**
A	593.88^a^	389.39^b^	418.20^b^	27.30	0.001	0.001	0.763
B	0.05	0.04	0.05	0.01	0.210	0.212	0.231
C	−0.05^a^	−0.06^a^	−0.13^b^	0.01	0.001	0.066	0.001
Lag time	1.34^c^	2.40^b^	5.34^a^	0.21	0.001	0.001	0.001
**Gas production**							
3h	22.56^a^	6.56^b^	2.61^b^	2.06	0.001	0.001	0.162
6h	70.32^a^	22.31^b^	10.43^b^	4.91	0.001	0.001	0.577
9h	157.89^a^	56.46^b^	28.69^b^	9.02	0.001	0.001	0.852
12h	282.63^a^	114.23^b^	65.21^b^	13.67	0.001	0.001	0.583
24h	367.57^a^	182.50^b^	182.62^b^	14.20	0.001	0.001	0.015
48h	497.66^a^	290.17^b^	336.58^b^	22.98	0.001	0.001	0.040
72h	553.38^a^	340.05^b^	374.42^b^	26.02	0.001	0.001	0.309
96h	635.64^a^	396.47^b^	426.60^b^	30.37	0.001	0.002	0.809
DMd	67.00^a^	54.67^b^	60.33^a^^b^	2.17	0.001	0.001	0.001
RGY	94.76^a^	73.15^b^	71.01^b^	41.85	0.004	0.102	0.005
GY24	73.51^a^	36.50^b^	36.52^b^	2.84	0.001	0.001	0.015

A, total gas produced expressed as ml gas/g DM; B (h^−1^) and C (h^−½^) are the gas production constants; DMd, dry matter disappearance expressed in g; RGY, relative gas yield expressed as ml gas/g DMd; GY24, gas yield production at 24 h expressed as ml gas 24h /g DMd; SEM, standard error of the mean.

a, b Different letters in the same column are different statistically (*p* < 0.05).

### Nutrient intake and digestibility

Nutrient intake and digestibility data are shown in Table 4. Dry matter and OM intake (g/kg LW^0.75^) were similar (*P* > 0.05) between treatments. Compared to GT200, ether extract was higher for GT0 and GT100 (*P* < 0.001). Compared with GT0, NDF intake was higher (*P* < 0.05) for GT200. Digestibility coefficients for DM, OM, NDF, and ADF were lower (*P* < 0.05) in GT100 than in the rest of the treatments.

**Table 4:** Dry matter intake (g/kg LW^0.75^, MBW) with different inclusion levels of whole green tomato silage (GT) as an alternative to corn silage in lamb diets and nitrogen balance (g/kg LW^0.75^, MBW).

**Variable**	**Treatment**			**SEM**	***P*** **-value**		
	**GT0**	**GT100**	**GT200**		**Treatment**	**Lineal**	**Quadratic**
MBW, kg	21.10	20.27	20.28	0.86	0.739	0.511	0.502
**Intake, MBW**							
DM	68.87	72.73	72.47	2.08	0.365	0.239	0.208
OM	60.92	63.15	61.98	2.03	0.742	0.715	0.448
CP	4.70^b^	5.43^a^	5.40^a^	0.17	0.013	0.011	0.008
EE	2.06^a^	2.02^a^	1.62^b^	0.06	0.001	0.001	0.599
NDF	32.03^b^	34.78^a^^b^	37.98^a^	1.13	0.008	0.002	0.188
ADF	14.78	15.40	15.11	0.49	0.685	0.641	0.392
ADL	3.68^b^	4.25^a^	4.56^a^	0.14	0.001	0.001	0.011
**Digestibility (g/kg)**							
DMd	650.0^a^	543.3^b^	606.7^a^^b^	22.7	0.015	0.197	0.004
OMd	660.0^a^	533.3^b^	605.0^a^^b^	22.8	0.004	0.108	0.001
NDFd	666.7^a^	523.3^b^	653.3^a^	8.4	0.002	0.615	0.001
ADFd	540.0^a^	353.3^b^	453.3^a^^b^	32.5	0.003	0.079	0.001
**Nitrogen balance (g/kg LW** ^**0.75**^ **, MBW)**							
N intake	0.75^b^	0.84^a^	0.86^a^	0.02	0.004	0.001	0.009
**N excreted**							
Feces	0.25^b^	0.39^a^	0.33^a^	0.02	0.001	0.016	0.001
Urine	1.72^b^	1.29^b^	3.10^a^	0.26	0.001	0.002	0.283
N balance	−1.22^a^	−0.86^a^	−2.37^b^	0.27	0.001	0.003	0.361

MBW, metabolic body weight; DM, dry matter; OM, organic matter; CP, crude protein; EE, ether extract; NDF, neutral detergent fiber; ADF, acid detergent fiber; ADL, acid detergent lignin; SEM, standard error of the mean.

a, b Different letters in the same column are different statistically (*p* < 0.05).

### Nitrogen balance

Nitrogen intake and nitrogen excreted in feces and urine (g/kg LW^0.75^) (Table 4), were lower (*P* < 0.001) for GT0. A negative nitrogen balance was also obtained in all treatments, and this had a linear effect (*P* < 0.001), being GT200 the one that has the most negative nitrogen balance (*P* < 0.001) compared with the rest of the treatments.

## Discussion

### Feed quality and diet composition

A proximate analysis of major feed ingredients is shown in Table 1. The DM, CP, NDF, and ADF contents of corn silage, alfalfa hay, soybean meal, canola meal and wheat bran indicated that these feeds are within the expected nutrient levels (10). There are no data related to the chemical composition of whole plant green tomato (as fresh or silage), however Fondevila et al. (19) reported data on tomato pomace (Solanum lycopersicum), which contains 24 % CP which is higher than whole plant green tomato silage (10.1%CP), however, they are similar in NDF content (56%).

Composition of the three diets fed in the study is shown in Table 2. Green tomato silage, which was added at the expense of corn silage, increased from about 6 to 12% of dietary DM (GT100 and GT200 respectively); actual CP concentrations ranged from 103 to 108 g/kg (DM basis). The inclusion of GT had little effect on NDF, ADF and Lignin content compared with the control diet.

### *In vitro* fermentation kinetics

*In vitro* gas production was lower for GT200. Besharati et al. (20) reported that in a study with red tomato addition, gas yields were lower in the initial incubation times compared to GT0 due to high contents of slowly fermented carbohydrates in tomato, as tomato has a high level of NDF (454 g NDF/ kg DM) and needs more time to adhere to the microorganism (21).

In this study, the decrease in gas production from tomato inclusion (GT100 and GT200) could be explained by flavonoid presence. Until now, not much information is available on the mechanism of action of flavonoids against rumen microbes, but flavonoids generally act against microorganisms by inhibiting cytoplasmic membrane function, inhibiting bacterial cell wall synthesis, or inhibiting nucleic acid synthesis (22, 23). Similarly, secondary compounds are inhibitors of gas production, ruminal microflora, protozoan content of ruminal flow, and proportionate production (24). In general, compared to control (GT0), green tomato treatments decreased fermentation parameters which agrees with studies showing that total gas production is negatively correlated with secondary plant metabolites (25).

The affected rumen fermentation kinetics observed in this study should be further considered in an experimental setup where less amounts of GT are added to diets and rumen microbiome is analyzed. Secondary plant compounds such as flavonoids, have antimicrobial effects and provoke a shift of protozoa and gram-positive bacteria populations in the rumen (26). Thus, using GT could be a nutritional strategy aiming at reducing enteric methane emissions not only in sheep but in other ruminant species. This approach has been recently revised (27–29) and dietary flavonoids have the potential for improving nutrient digestibility and animal performance.

### Nutrient intake and digestibility

The observed increase in NDF and lignin intake observed with GT100 and GT200 could be attributed to a higher amount NDF content of tomato plants, compared with CS, which is associated with the phenological stage of the plant, which in this study was in its final growth stage (stem and green tomatoes). A limiting factor that affects the efficiency of energy use is the excess of fiber, which limits the development of the animals. Diets with high amounts of indigestible fiber can lead to low production of volatile fatty acids which eventually affects energy synthesis and availability (30, 31).

Dry matter and OM digestibility were similar among treatments, on the contrary, NDF and ADF digestibility decreased as the GT inclusion increased, contrary to Abdollahzadeh et al. (32) and Gawad et al. (33), who reported that feeding tomato silage as a replacer for alfalfa hay for Holstein dairy cows and buffaloes, led to a significant increase in the digestibility of CP and NDF. The lower digestibility of NDF with respect to the inclusion of GT may be because it has a higher concentration of indigestible NDF and lignin.

The fiber composition of the basal diet used to feed sheep is important as it could lead to different effects on OM digestibility, for example, it has been reported that OM digestibility of dried tomato from 56% (12) in lambs fed on alfalfa hay-based diets while lambs fed on dried tomato in a diet based on barley straw can have an OM digestibility of 90% (3, 34).

### Nitrogen balance

In the present study, animals had a negative nitrogen balance, however, sheep fed on GT200 had the highest nitrogen loss compared with GT0 and GT100. This could be due to a higher presence of secondary metabolites including phenolic compounds, phytoalexins, protease inhibitors, and glycoalkaloids in green tomatoes, which could have decreased the efficiency of N utilization, making it difficult to absorb and usage of N in the digestive tract (30, 35, 36), and consequently, the nitrogen content was lost, resulting in the microbial protein in the post-ruminal tract not reflected into sheep growth (37). Also, tomato plants (green or red) synthesize glycoalkaloids dehydrotomatine and alpha-tomatine, as a defense against bacteria, fungi, viruses, and insects (38). Yamashoji and Onoda (39), evaluated the antiobesity effect of immature green tomatoes and reported that they inhibited the accumulation of lipid in adipocytes, a-tomatine from tomatoes interferes with cholesterol absorption and increase sterol excretion by forming a non-absorbable complex with cholesterol in the gastrointestinal tract. Health hazards of a-tomatine have been studied by various animal tests and it is known that the toxicity of α-tomatine depends on the presence of lycotetraose, because removal of one or all four sugar residues renders α-tomatine less toxic (40). In this study, it is possible that tomato secondary compounds disrupted rumen microorganisms' populations with negative effects on nutrient metabolism. Further studies should consider analyzing rumen microbiome to confirm these findings.

The reported apparent digestibility of N from dried tomato pomace was less than that from soybean meal (41). A highly concentrated diet containing dried tomato at 30% of the DM had an apparent CP digestibility of 51% compared with 69% for a diet containing soybean meal (33, 41). Previous reports on the N balance of lambs fed dried tomato pomace are not consistent. In agreement with our data, Ammerman et al. (41) reported a lower N balance when lambs were fed with dried red tomato than when lambs were fed with soybean meal. However, Fondevila et al. (19) reported no difference in N balance between lambs fed dried red tomato and that fed soybean meal. In the present study, urine excretion was highest for GT200 (2,004 mL), followed by GT100 (1,139 mL) and the lowest excretion was for GT0 (569 mL). Increases in urine volume in animals fed green tomatoes increased urinary N excretion, with GT200 having the most negative nitrogen balance (Table 4) with a 94 % increase in total N excretion with respect to GT0. This increase in the excretion of urine and therefore of N, can be seen as a mechanism of excretion of toxic compounds present in tomato (α-tomatine) (42) and consequently, a greater excess of N in urine in the animal by including GT00 and GT200.

This increase in urine excretion was reflected in an increase in water consumption, possibly due to a detoxification response of the animals as it has been observed that the green tomatoes whole plant accumulates a variety of secondary metabolites (38, 43). In this sense, it has been reported that dehydrotomatine and alpha-tomatine contents from tomatoes varied from 42 to 1,498 and 521 to 16,285 mg/g of fresh weight, respectively (42, 43), which represents 120 times more tomatine than red tomatoes (38). Taken together, in this study, it is very possible that this increase in tomatine affected animal welfare, and consequently, one way to eliminate the compound was by increasing the excretion of urine and therefore of *N*.

## Conclusions

In sheep diets, the inclusion of whole plant green tomato silage at 100 or 200 g/kg DM as an alternative to corn silage, does not affect dry matter intake but negatively affects nutrient digestibility and N balance. Therefore, under the conditions of this study, its inclusion in sheep diets is not recommended.

## Data availability statement

The raw data supporting the conclusions of this article will be made available by the authors, without undue reservation.

## Ethics statement

The animal study was reviewed and approved by all experimental procedures were approved by the Animal Experimental Guidelines of the Universidad Autónoma del Estado de México (Project Code: UAEMex 4974/2020).

## Author contributions

LER-J, MG-R, and EV-B-P are the principal investigator of this project. ACN-L, MG-R, and EV-B-P conducted the experiment. ACN-L, AS-R, and AJC-C conducted the data analysis. LER-J, OAC-O, NZ, MG-R, and EV-B-P drafted the manuscript. All authors contributed to the article and approved the submitted version.

## Funding

This project was partially supported by Universidad Autónoma del Estado Mexico (Grant Number: UAEMex 4974/2020).

## Conflict of interest

The authors declare that the research was conducted in the absence of any commercial or financial relationships that could be construed as a potential conflict of interest.

## Publisher's note

All claims expressed in this article are solely those of the authors and do not necessarily represent those of their affiliated organizations, or those of the publisher, the editors and the reviewers. Any product that may be evaluated in this article, or claim that may be made by its manufacturer, is not guaranteed or endorsed by the publisher.
